# The Use of Computer-Assisted Home Exercises to Preserve Physical Function after a Vestibular Rehabilitation Program: A Randomized Controlled Study

**DOI:** 10.1155/2016/7026317

**Published:** 2016-02-11

**Authors:** Michael Smaerup, Uffe Laessoe, Eric Grönvall, Jens-Jacob Henriksen, Else Marie Damsgaard

**Affiliations:** ^1^Department of Geriatrics, Aarhus University Hospital, 8000 Aarhus C, Denmark; ^2^Department of Physiotherapy/Research and Development, University College of North Denmark, 9220 Aalborg, Denmark; ^3^Computer Games and Interaction Design, IT University of Copenhagen, 2300 Copenhagen, Denmark; ^4^Department of Ear, Nose and Throat, Aarhus University Hospital, 8000 Aarhus C, Denmark

## Abstract

*Objective.* The purpose of this study was to evaluate whether elderly patients with vestibular dysfunction are able to preserve physical functional level, reduction in dizziness, and the patient's quality of life when assistive computer technology is used in comparison with printed instructions.* Materials and Methods*. Single-blind, randomized, controlled follow-up study. Fifty-seven elderly patients with chronic dizziness were randomly assigned to a computer-assisted home exercise program or to home exercises as described in printed instructions and followed for tree month after discharge from an outpatient clinic.* Results*. Both groups had maintained their high functional levels three months after finishing the outpatient rehabilitation. No statistically significant difference was found in outcome scores between the two groups. In spite of moderate compliance levels, the patients maintained their high functional level indicating that the elderly should not necessarily exercise for the first three months after termination of the training in the outpatient clinic.* Conclusion*. Elderly vestibular dysfunction patients exercising at home seem to maintain their functional level, level of dizziness, and quality of life three months following discharge from hospital. In this specific setup, no greater effect was found by introducing a computer-assisted training program, when compared to standard home training guided by printed instructions. This trial is registered with NCT01344408.

## 1. Introduction

Dizziness is characterized by postural instability, disequilibrium, and poor spatial orientation [1]. It is a problem in the elderly population, with a reported prevalence of 11% to 39% and a significant increase with age [1–3]. Improvement following vestibular rehabilitation (VR) is well documented regardless of age or gender, and VR is an effective intervention in people with chronic vestibular disorders [4–6]. Since older adults exhibit less exercise compliance than others [7], it seems relevant to study the effectiveness of VR in elderly patients with vestibular dysfunction after discharge from hospital rehabilitation.

The issue concerning VR is whether improvements persist after the supervised training in the outpatient clinic. A review that assessed the effectiveness of VR in community-dwelling adults confirmed that any positive effect obtained was maintained for three to twelve months [8]. However, the studies did not focus on patients aged 65 years or older. A review [9] examining the effects of VR in middle-aged and elderly adults described one study with a follow-up period of three months where the intervention group preserved significant improvement in the one-leg stand test in comparison to the control group. Another study in the review examining the effects of VR six months and one year after the intervention found that intervention and control group reached their previous functional levels and maintained the gain obtained in the period [10]. The first study included patients with central vestibular dysfunction and dizziness of age-related origin. The study may be biased as the patients were diagnosed by a general practitioner and not by a trained otoneurologist. The other study included only patients with acoustic neuroma and the results are not transferable to elderly vestibular patients with various vestibular diagnoses.

Studies show that the greatest drop in exercise compliance occurs at the end of the training program in an outpatient clinic and at the same time the long-term adherence to home exercises seems low [11, 12]. This makes it relevant to examine whether home exercise may be optimized. “Exergames” [13] (exercise + gaming) may be promising for home-based balance and strength training of the healthy elderly and have several advantages compared to conventional exercise, since exergaming seems to motivate people to practice. The review showed that the number of controlled studies examining graphical games is small, but existing studies report a high degree of enjoyment and motivation to perform such exercises [13]. Based on this knowledge, it seemed relevant to test exergames in a home setting among older adults with chronic vestibular dysfunction since lifelong VR often is needed to decrease vestibular symptoms. However, we do not know whether it is possible to maintain or improve the functional level obtained through a computerized exercise program without supervision of a physiotherapist. In addition we found great variation in compliance rates in computer-based intervention studies focused on the elderly. Gschwind et al. [14] found a low compliance level (14%) with an in-home intervention with Microsoft-Kinect. Schoene et al. [15] found a high compliance level of 92% with an in-home intervention by videogame technology.

The purpose of this study was to evaluate whether elderly patients with vestibular dysfunction are able to preserve their physical function level, reduction in dizziness, and quality of life with assistive computer technology in comparison to printed instructions.

## 2. Methods

The study was performed as an extension of an RCT study of computer-assisted training in a hospital-based, supervised vestibular rehabilitation program and was conducted from January 2010 to July 2013 [16]. After discharge from hospital, the intervention group continued with computer-assisted training in a home setting and the control group with printed instructions for home exercises which was the standard of care. Only participants who completed the hospital rehabilitation program were included in this study. An assessor blinded to the exercise program examined all participants at the termination of the supervised training in the outpatient clinic and again 12 weeks later.

Findings in a rehabilitation exercise trial in patients with dizziness showed a mean improvement of 6.6 ± 8.4 seconds on the one-leg stand test in the intervention group compared with 0.4 ± 6.9 seconds in the control group [17]. In our previous study [18] we expected a mean improvement of approximately 6 seconds for the intervention group compared with the control group. On the assumption of 2-tailed significance of 5%, 80% power, and an expectation of 15% dropouts, it was estimated that the sample size should be 29 patients per rehabilitation group.

The measurements were recorded during one-hour sessions by a blinded assessor:* one-leg stand test* was used to measure postural control [18] and was the primary endpoint. In standing on one leg, the time was recorded until the subject moved his feet from the original position or reached the maximum time of 30 seconds.* Dynamic Gait Index* was used to assess dynamic postural stability [19]. The test consists of eight functional tasks scored on a four-level ordinal scale from 0 to 24 points.* Dizziness Handicap Inventory* [20] was used to measure the impact of dizziness on the quality of life on a range score from 0 to 100 points.* Short Form-12* was used to assess quality of life [21] and the response is presented as both a Physical Composite Score and a Mental Composite Score with a range from 0 to 100 points.* Motion Sensitivity Test* was used to measure motion-promoted dizziness during a series of 16 rapid changes of head or body position with a range of 0 to 128 points [22].* Visual Analogue Scale* [17] was used to rate the participant's vertigo on a scale from 0 to 100 mm (from no symptoms to the worst possible vertigo).* The Chair Stand Test* [23] was used to measure strength of the lower extremities by recording the number of times the participant manages to rise from a chair within 30 seconds.

The Danish National Committee on Health Research Ethics (project ID: M-20090189) and The Danish Data Protection Agency (project ID: 1-16-02-84-09) approved this study.

### 2.1. Participants

All participants in the study gave signed and informed consent. Inclusion criteria were 65 years of age or older and stable peripheral, central, or mixed vestibular dysfunction. The participants were recruited from the Department of Geriatrics, Aarhus University Hospital, Denmark. A geriatrician evaluated the causes for the patients' falls. Patients with vestibular dysfunction who agreed to participate in the project were referred to the Ear, Nose, and Throat Department at Aarhus University Hospital for confirmation of their diagnoses [16].

Exclusion criteria were unstable peripheral vestibular dysfunction including Ménière's disease, Benign Paroxysmal Positional Vertigo (BPPV), and acute vestibular neuronitis. Other exclusion criteria were severely impaired eyesight (6/60 or less), significant cardiac problems, use of medication with risks of vestibular side effects (benzodiazepines, sedatives), dementia (mini-mental state examination <27 or a history suggesting dementia), stroke within the preceding six months, other cognitive dysfunctions, and hip fracture within the preceding three months.

### 2.2. Randomization

**Figure 1:**
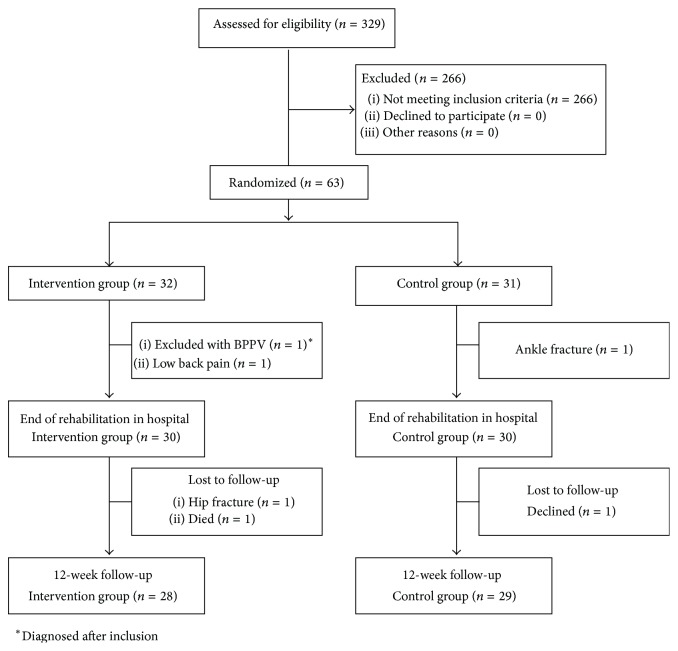
Flow chart.

In the hospital-based study that preceded the present study, the randomization was provided by a central computer program using permuted block sizes and stratification, according to peripheral, central, or mixed vestibular dysfunction [16]. The sample size in our previous study was estimated to 29 patients per rehabilitation group [16]. The present follow-up study includes 28 patients assigned to intervention and 29 patients in the control group (Figure 1).

### 2.3. Intervention

The computerized training program, “Move It To Improve It” (Mitii) [24] was installed in the participant' homes using an internet-connected computer with a web camera connected to a cloud-based specifically adapted interactive training program. A sequence of individual games was arranged for a daily exercise program of 20 to 30 minutes with the patient in a standing position. Before each game, a short video showed the patient what to do. The program comprised drag-and-drop and follow-the-leader games. For drag-and-drop games, patients wore a headband with a green marker at the front. The webcam registered the position of the marker and transferred this information to the screen cursor control to be controlled by head movements. A virtual object on the screen was manipulated by grabbing and dragging it to a different location or onto another virtual object. A follow-the-leader game uploaded a video sequence of the therapist's movements that the patient was expected to follow visually. These games challenged the patient's vestibule-ocular reflex and postural control. After completing each game, a “well done” appeared at the screen. No other feedback was given concerning the manner or quality of performance, but the duration was registered and displayed for the hospital project physiotherapist who contacted participants if the program was not used for seven days.

The training in the control and intervention group aimed at vestibular-ocular and cervical-ocular reflex training for gaze stability, resetting of vestibular-ocular reflex gain, enhancing smooth-pursuit eye movements, and the ability to utilize somatosensory and vestibular input for postural control [16].

A physiotherapist emphasized to all participants the importance of continuing the exercise program at least once daily to maintain the functional level after completing rehabilitation in the outpatient clinic and continue their home exercise program without contact to a physiotherapist. The duration of the exercise program sessions was between 20 and 30 minutes for both groups.

Compliance data in the intervention group were measured online by the Mitii program when the patients logged on the system.

### 2.4. Statistical Analysis

The data were analysed using STATA statistical software version 12. Baseline in this study was defined as the point of discharge of the supervised training in the outpatient clinic. An independent *t*-test was used to compare baseline parameters between the two groups. In each group, the outcomes measured at baseline and at 12-week follow-up were compared with a paired *t*-test (within group test). Furthermore, the groups were compared with a two-sample independent *t*-test with respect to the change from baseline to 12-week follow-up (within group test). As some of the variables showed departures from the normal distribution, we also compared data with nonparametric Wilcoxon signed rank tests, but these analyses did not change the conclusions.

Compliance in the intervention group was calculated by dividing the number of performed training sessions by the number of recommended training sessions. Twelve weeks of daily home exercise corresponds to 84 recommended sessions for each participant. The Wilcoxon signed rank test was used to analyse the change in compliance during the 12 weeks of home exercise. The association between time spent on training at home and the change in measured outcome was tested using Spearman's rank correlation.

## 3. Results

The participants in the intervention and control group did not differ at baseline for the home training period (*p* > 0.05 for all variables, see Table 1). The mean duration of dizziness among the participants was more than five years. Central vestibular dysfunction was the most common diagnosis.

### 3.1. Computer-Assisted Home Training Program versus Printed Instructions

We did not observe any significant difference in functional level, level of dizziness, or quality of life between the groups (Table 2).

### 3.2. Exercise Compliance in the Intervention Group

The participants in the intervention group used the Mitii system in a mean of 33 of the 84 possible days (41%) of the home training period (median: 30 sessions, 25th percentile = 0 sessions, and 75th percentile = 49 sessions). The time spent on the at-home training sessions was not associated with outcome when tested by Spearman's rank correlation.

**Figure 2:**
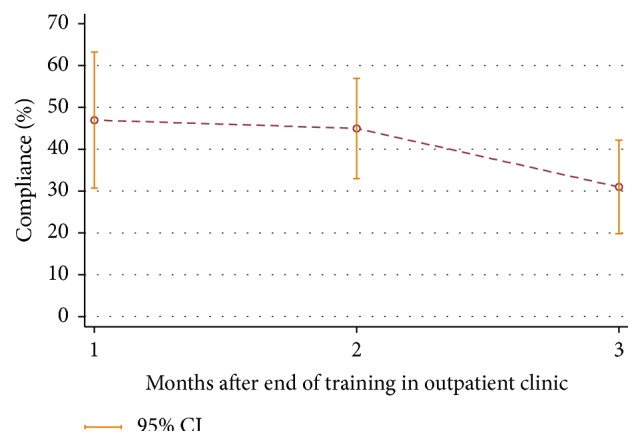
Compliance in the home training period in the intervention group. CI, confidence interval.

A reduction in training compliance was seen over the period from the first to the third month of home training (Figure 2). Testing with the Wilcoxon signed rank test showed a significant decrease in compliance with period from month two to month three (*p* < 0.001).

## 4. Discussion

### 4.1. Functional Level Three Months after Completion of Supervised Training

Our findings indicate that elderly patients trained in an outpatient clinic for vestibular dysfunction are able to maintain functional level in up to three months. Our results are confirmed by Yardley et al. [25] who included patients older than 60 years with vestibular dysfunction. At the six-month follow-up, the study found that improvement obtained during a three-month home exercise program delivered by nurses was maintained in the intervention group. Unfortunately the study could not present the measures in the control group receiving usual medical care since the design was a crossover (the controls were instructed in home exercises after three months). Hansson et al. [26] included vestibular patients with a median age of 77 years and found statistically significant differences between control group (sham group) and intervention group (group sessions in a physiotherapy centre for six weeks). Statistically significant differences were found between the two groups, comparing the results at baseline and after six weeks on the one-leg stand test with eyes closed. After three months, the difference between the groups was statistically significant with an improvement in the intervention group and deterioration in the control group.

In Cohen and Kimball's study [27], the vestibular patients were randomly assigned to three home program treatment groups: (1) slow head movements while seated, (2) rapid head movements while seated and while standing, and (3) rapid head movements. They received a weekly telephone call to encourage compliance. All subjects performed home programs for four weeks. The study showed that the Dizziness Handicap Inventory score decreased (improved) from pretest to posttest and then continued to decline over a 6-month follow-up period for all three groups. The mean age of the patients included was 51.1 years. This could be the explanation for the improvement in the study compared to ours as other studies have concluded [28] that a low level of physical activity at baseline (like the elderly people in our study) is a barrier for treatment compliance.

### 4.2. The Computer-Assisted Home Training Program versus Printed Instructions

We did not find any significant difference in functional levels three months after the end of hospital training between patients instructed in a printed home training program and those having a computer-assisted training program indicating that exercising within the first three months after termination of the training in the outpatient clinic is not necessary among elderly patients.

No previous study has investigated the difference between printed versus computer-assisted home training programs among vestibular patients. The closest is the study by Pavlou et al. [29] who compared a customized vestibular exercise program (both clinic training and printed instructions at home) with a simulator based regime (therapeutic stimulation at the clinic and video stimulation at home). Pavlou et al. found that customized exercises both with and without simulator based exposure improve subjective symptoms, postural stability, and emotional status in chronic vestibular patients.

There are several possible explanations for the lack of difference between the intervention and control groups in our study. The simplest explanation is that Mitii could not motivate the elderly and thus is not suitable for this population. Conversely, in a 2013 review [12] McLean et al. showed that the elderly found training with exercise games more appealing than traditional training. They were more motivated to exercise and showed greater improvements in measured clinical outcomes than did the controls. However, these studies took place in a clinical setting with physiotherapists to motivate the participants.

Another explanation for the missing difference between control and intervention groups could be due to a possible ceiling effect on the outcome measures since the patients' scores at baseline on the Dynamic Gait Index, Dizziness Handicap Inventory, and Motion Sensitivity Test were close to maximum. Since the patients from the start of the study had this high level, it is difficult to demonstrate an improvement, with time. Sluijs et al. showed that patients will be more compliant if they believe that exercises contribute to recovery [28].

In this study, the compliance in the intervention group was 41%, making the intervention moderately adopted. Surprisingly, the compliance level seemed high enough to maintain the functional level, quality of life, and the reduced dizziness level among the patients. We still need some knowledge about the compliance in the control group but it seems that the printed instructions were as effective as Mitii. As previously mentioned, a possible explanation why we could not measure an effect of Mitii could be that the patients had high functional levels when the study started and the follow-up time was too short for observing a change in function among the patients. Maybe a follow-up time of one year could identify if Mitii could increase compliance in the long term. This would be relevant to the elderly vestibular patients who may need lifelong training [30].

Another explanation for the missing difference in the two training groups may be that we, like other VR training studies, could not produce a protocol with a sham exercise group or waiting list patients as controls, since we could not defend not including the patients immediately to vestibular rehabilitation since they all were elderly people at risk of falling.

In one of the few VR studies measuring compliance, Yardley and Kirby [31] compared two intervention groups with a waiting list control group. The study showed that both intervention groups reported greater compliance than controls. However, it is to be expected that waiting list controls and patients at the offset of a training period must be more motivated for exercising than patients who have completed a rehabilitation program in an outpatient clinic. This is supported by prior studies that found that compliance was highest at the beginning of a period of training and decreased over time [29, 32].

## 5. Limitations

Participants and therapists administering rehabilitation could not be blinded. An attempt was made to blind assessors measuring the outcomes, but the measurements were taken during one-hour sessions, so a risk of revealing the patients' treatment group was introduced.

Another limitation is that we did not measure how the participants accepted the different modes of home exercise delivery. This might have provided valuable information for future studies aiming at effective interventions by home exercise programs.

## 6. Conclusion

Elderly vestibular dysfunction patients exercising at home seem to maintain functional level obtained through supervised vestibular training in an outpatient clinic.

In this specific setup, there were no significant differences between computer-assistance and printed instructions guidance of those leaving outpatient rehabilitation programs, and thus provision of complex computer systems does not appear necessary.

The elderly patients may be at a high functional level at the end of their outpatient clinic training period and therefore may not see the relevance of continuing training guided by printed instruction or assistive technology.

## Figures and Tables

**Table 1:** Participant characteristics at baseline, that is, termination of supervised training in outpatient clinic^*∗*^.

Variables	Mitii group (*n* = 28)^a^	Control group (*n* = 29)
Women, *n* (%)	17 (57)	19 (63)
Age	76.39 ± 7.63	78.93 ± 6.58
Duration of dizziness, months	61.50 ± 52.25	69.66 ± 47.38
Type of vestibular dysfunction, *n* (%)		
Peripheral	2 (7)	2 (7)
Mixed	4 (14)	6 (21)
Central	22 (79)	21 (72)
One-leg stand test (s)	11.90 ± 10.61	11.11 ± 10.66
Dynamic Gait Index (points)	17.68 ± 4.20	16.41 ± 3.89
Dizziness Handicap Inventory (points)	31.36 ± 19.78	35.27 ± 18.08
Motion Sensitivity Test (points)	15.43 ± 15.72	18.07 ± 22.00
Visual Analogue Scale (mm)	29.89 ± 2.06	30.17 ± 19.38
Chair Stand Test (rep)	13.00 ± 4.50	12.17 ± 2.88
Short Form-12 Physical Composite Score (points)	41.92 ± 13.12	38.91 ± 11.61
Short Form-12 Mental Composite Score (points)	56.05 ± 8.57	53.83 ± 9.45

^*∗*^Values with a plus/minus sign are means ± SD.

^a^The intervention with a computerized training program.

**Table 2:** Changes during intervention period^a^.

Measure^b^	Mitii group^c^		Control group		Difference between groups	
	Change during intervention period mean (95% CI) Paired *t*-test	*p* value	Change during intervention period mean (95% CI) Paired *t*-test	*p* value	Difference intervention period mean (95% CI) Independent *t*-test	*p* value
One-leg stand test (s)	0.41 (−1.34 to 2.15)	0.54^*∗*^	1.66 (−0.61 to 3.93)	0.18^*∗*^	−1.26 (−4.07 to 1.56)	0.38^*∗*^

Dynamic Gait Index (points)	0.07 (−0.79 to 0.93)	0.86	−0.28 (−1.06 to 0.51)	0.48	−0.35 (−1.48 to 0.78)	0.54

Dizziness Handicap Inventory (points)	1.64 (−1.76 to 5.05)	0.33	0.97 (−3.78 to 5.71)	0.68	−0.67 (−6.43 to 5.07)	0.81

Motion Sensitivity Test (points)	2.11 (−1.79 to 6.00)	0.15^*∗*^	−1.52 (−8.50 to 5.47)	0.41^*∗*^	−0.26 (−4.20 to 3.68)	0.12^*∗*^

Visual Analogue Scale (mm)	−3.29 (−9.10 to 2.53)	0.35^*∗*^	−2.76 (−11.18 to 5.66)	0.48^*∗*^	0.53 (−9.51 to 10.56)	0.92

Chair Stand Test (rep)	−0.54 (−1.28 to 0.21)	0.17	−0.03 (−1.02 to 0.95)	0.86	0.50 (−0.71 to 1.72)	0.41

Short Form-12 Physical Composite (points)	1.11 (−2.68 to 4.91)	0.95^*∗*^	1.79 (−2.04 to 5.61)	0.27^*∗*^	−1.46 (−4.07 to 1.16)	0.58

Short Form-12 Mental Composite (points)	−2.19 (−4.54 to 0.16)	0.08^*∗*^	−0.74 (−4.73 to 3.26)	0.14^*∗*^	1.45 (−3.10 to 6.01)	0.99^*∗*^

^a^Analyses are based on data from *n* = 28 in the intervention group and *n* = 29 in the control group.

^b^Positive mean values indicate a better function, except for the Motion Sensitivity Test, Dizziness Handicap Inventory, and Visual Analogue Scale.

^c^The intervention with a computerized training program.

^*∗*^Wilcoxon Rank-Sum test.
